# Adapting SureSelect enrichment protocol to the Ion Torrent S5 platform in molecular diagnostics of craniosynostosis

**DOI:** 10.1038/s41598-020-61048-5

**Published:** 2020-03-05

**Authors:** Ewelina Bukowska-Olech, Delfina Popiel, Grzegorz Koczyk, Anna Sowińska-Seidler, Magdalena Socha, Bartosz Wojciechowicz, Adam Dawidziuk, Dawid Larysz, Aleksander Jamsheer

**Affiliations:** 1 Department of Medical Genetics, Poznan University of Medical Sciences, Rokietnicka 8 Street, 60-806 Poznan, Poland; 2 Postgraduate School of Molecular Medicine, Medical University of Warsaw, Żwirki i Wigury 61 Street, 02-091 Warsaw, Poland; 3 Centers for Medical Genetics GENESIS, Grudzieniec 4 Street, 60-601 Poznan, Poland; 4 Department of Biometry and Bioinformatics, Institute of Plant Genetics, Polish Academy of Sciences, Strzeszyńska 34, 60-479 Poznań, Poland; 5 Perlan Technologies Sp. z o.o., Puławska 303 Street, 02-785 Warsaw, Poland; 6 Department of Radiotherapy, The Maria Skłodowska Curie Memorial Cancer Centre and Institute of Oncology, Gliwice Branch, 44-101 Gliwice, Poland

**Keywords:** Clinical genetics, Development

## Abstract

Obtaining reliable and high fidelity next-generation sequencing (NGS) data requires to choose a suitable sequencing platform and a library preparation approach, which both have their inherent assay-specific limitations. Here, we present the results of successful adaptation of SureSelect hybridisation-based target enrichment protocol for the sequencing on the Ion Torrent S5 platform, which is designed to work preferably with amplicon-based panels. In our study, we applied a custom NGS panel to screen a cohort of 16 unrelated patients affected by premature fusion of the cranial sutures, i.e. craniosynostosis (CS). CS occurs either as an isolated malformation or in a syndromic form, representing a genetically heterogeneous and clinically variable group of disorders. The approach presented here allowed us to achieve high quality NGS data and confirmed molecular diagnosis in 19% of cases, reaching the diagnostic yield similar to some of the published research reports. In conclusion, we demonstrated that an alternative enrichment strategy for library preparations can be successfully applied prior to sequencing on the Ion Torrent S5 platform. Also, we proved that the custom NGS panel designed by us represents a useful and effective tool in the molecular diagnostics of patients with CS.

## Introduction

## Next-Generation Sequencing (NGS) in Medical Genetics

Routine NGS diagnostics requires high-quality sequencing data, short turnaround time and reasonable cost of the investigations. Therefore, out of the three major NGS-based diagnostic strategies, i.e. whole exome sequencing (WES), whole genome sequencing (WGS), and targeted gene panel sequencing, the final approach is ubiquitous and broadly applied in the clinical settings^1–5^. Successful implementation of targeted NGS in medical diagnostics results from several advantages. First, it generates disease-restricted data with fewer variants of uncertain significance, simplifying the analysis. Next, it provides very high coverage and read depth of selected regions, and finally, it limits the need for expensive laboratory equipment and data storage^6,7^. In order to generate reliable, high fidelity NGS data one has to choose a suitable sequencing platform and a library preparation protocol^6,8^. Different NGS platforms are known to have their specific limitations, such as underrepresentation of sequences with high guanine-cytosine (GC) content in case of Illumina or homopolymer length estimation bias in Ion Torrent semiconductor-based sequencing systems^9–14^. In addition to dissimilarities of NGS platforms and their specific inbuilt artefacts, also the sample preparation protocols differ in many aspects, including enrichment strategy. Currently, two major targeted enrichment strategies are available, i.e. PCR-based methods and hybridisation-based protocols. Although targeted PCR-based amplicon approach offers both easy workflow and shorter reaction time, requiring low DNA input at the same time, it suffers from several limitations, such as lower sequencing complexity and coverage uniformity^15–18^. In general, the problem of non-specificity in PCR-based methods often cannot be circumvented by careful primer design, as the oligonucleotides have usually very short sequence^16,18,19^. On the other hand, an alternative approach, i.e. hybridisation-based enrichment protocols such as SureSelect (Agilent Technologies) is available and is commonly applied on the Illumina platforms. The SureSelect approach is based on biotinylated RNA oligomers of substantially greater length (120 bp), which can bind to DNA more specifically and consequently enrich the targeted regions of the genome, avoiding repetitive or non-specific amplification. Therefore, SureSelect enrichment strategy allows for obtaining better sequencing complexity and coverage uniformity^16^.

To our knowledge, SureSelect libraries have not been used so far to carry out the sequencing on the Ion Torrent S5 semiconductor-based platform. In this report, we present the first example of a successful adaptation of the hybridisation-based SureSelect enrichment protocol to the sequencing on the Ion Torrent S5 system. In addition, using a cohort of patients presenting with craniosynostosis, we emphasise the utility of targeted gene panel sequencing in the diagnostics of this aetiologically heterogeneous condition.

## Craniosynostosis

Craniosynostosis (CS), premature fusion of one or more cranial sutures, occurs either as an isolated malformation or in a syndromic form, representing a genetically heterogeneous and clinically variable group of disorders^20^. Routine diagnostic screening of common craniosynostosis-associated genes (usually *FGFR1*, *FGFR2*, *FGFR3*, *TWIST1* and often *EFNB1*, *TCF12*) enables to establish genetic aetiology in 21%^21^ to 62%^22^, depending on the size of the study, ethnicity of the population, and range of the molecular analysis (either hot-spot screening or the entire gene sequencing). Since targeted NGS is regarded as a useful diagnostic method in identification of causative variants, especially in genetically heterogeneous diseases^23,24^, we have designed and applied a custom hybridisation-based panel (Agilent Technologies) to screen CS patients with negative results of preliminary molecular screening (involving hot-spot mutations located in exon 7 of *FGFR1*, exons 7 and 8 of *FGFR2*, and exon 7 of *FGFR3*, as well as we analysed the entire coding sequence of *TWIST1*).

## Results

### Clinical description

We used an NGS targeted gene panel approach to screen 16 consecutive patients with CS in whom the result of conventional Sanger sequencing of preliminary molecular screening was negative. Distribution of prematurely fused sutures was as follows: coronal – 6/16 (unilateral – 4, bilateral – 2), metopic – 5/16, sagittal – 3/16, multiple – 2/16. 56.25% of patients from our cohort presented with the syndromic form of CS, whereas 43.75% had an isolated defect. All patients were subjected to a careful dysmorphological assessment upon which clinically recognisable craniofacial malformations and other defects were photographically documented. Additionally, diagnostic imaging, including X-rays, CT scans, or head MRI was performed. DNA was extracted from venous blood samples of index patients and their parents.

### Custom gene panel

On the basis of clinical reports available in medical literature and databases (OMIM, MGI) we have chosen the gene and variant content and designed a hybridisation-based panel comprised of 61 genes (see Table 1) and 11 SNVs (see Table 2) thought to be associated with craniosynostosis and abnormalities of craniofacial development. To create our gene panel we have used SureDesign software (Agilent Technologies, SantaClara, USA). The designed panel was further refined in collaboration with Perlan Biotechnologies. The panel summary is as follows: Agilent Design ID: 3056721, panel name: Cranio_V1, region size: 173.794 kb, 6033 probes (225 709 kb) with region extension: 25 bases from 3′ end and 25 bases from 5′ end. The panel was classified to price tier 1, in which target region size ranges from 1 to 499 kbs. Hence, the target sequence and also the gene content could be increased at least two times without additional cost.

**Table 1:** Genes included in craniosynostosis-associated custom panel.

Gene	HGNC ID	Reference sequence number	Disorder (#OMIM)	Mode of inheritance	Inclusion support
*ALPL*	438	NM_000478	Different forms of hypophosphatasia	AR	Clinical evidence (OMIM, Pubmed)
*ALX1*	1494	NM_006982	Frontonasal dysplasia 3 (613456)	AR	Clinical evidence (OMIM, Pubmed)
*ALX3*	449	NM_006492	Frontonasal dysplasia 1 (136760)	AR	Clinical evidence (OMIM, Pubmed)
*ALX4*	450	NM_021926	Frontonasal dysplasia 2 (613451),	AR	Clinical evidence (OMIM, Pubmed)
			Parietal foramina 2 (609597),	AD	
			{Craniosynostosis 5, susceptibility to} (615529)	AD	
*BMP4*	1071	NM_001202	Craniofacial development		Literature review (Pubmed, MGI)
*CYP26B1*	20581	NM_019885	Craniosynostosis with radio humeral fusions and other skeletal and craniofacial anomalies (614416)	AR	Clinical evidence (OMIM, Pubmed)
*DHODH*	2867	NM_001361	Miller syndrome (263750)	AR	Clinical evidence (OMIM, Pubmed)
*DPH1*	3003	NM_001383	Developmental delay with short stature, dysmorphic features, and sparse hair (616901)	AR	Clinical evidence (OMIM, Pubmed)
*EDN3*	3178	NM_207034	Craniofacial development		Literature review (Pubmed)
*EDNRB*	3180	NM_000115	Craniofacial development	AD, AR	Literature review (MGI, Pubmed)
*EFNA4*	3224	NM_005227	Nonsyndromic coronal craniosynostosis	AD?	Literature review (OMIM, Pubmed)
*EFNB1*	3226	NM_004429	Craniofrontonasal syndrome (304110)	XD	Clinical evidence (OMIM, Pubmed)
*EFTUD2*	30858	NM_004247	Mandibulofacial dysostosis, Guion-Almeida type (603892)	AD	Clinical evidence (OMIM, Pubmed)
*ERF*	3444	NM_006494	Craniosynostosis 4 (600775)	AD	Clinical evidence (OMIM, Pubmed)
*ESCO2*	27230	NM_001017420	Roberts syndrome (268300)	AR	Clinical evidence (OMIM, Pubmed)
*FGFR1*	3688	NM_023110	Hartsfield syndrome (615465)	AD	Clinical evidence (OMIM, Pubmed)
			Jackson-Weiss syndrome (123150)		
			Osteoglophonic dysplasia (166250)		
			Pfeiffer syndrome (101600)		
			Trigonocephaly 1 (190440)		
*FGFR2*	3689	NM_000141	Antley-Bixler syndrome without genital anomalies or disordered steroidogenesis (207410)	AD	Clinical evidence (OMIM, Pubmed)
			Apert syndrome (101200)		
			Beare-Stevenson cutis gyrata syndrome (123790)		
			Bent bone dysplasia syndrome (614592)		
			Craniofacial-skeletal-dermatologic dysplasia (101600)		
			Crouzon syndrome (123500)		
			Jackson-Weiss syndrome (123150)		
			LADD syndrome (149730)		
			Pfeiffer syndrome (101600)		
			Saethre-Chotzen syndrome (101400)		
			Scaphocephaly, maxillary retrusion, and mental retardation (609579)		
*FGFR3*	3690	NM_000142	Achondroplasia (100800) Crouzon syndrome with acanthosis nigricans (612247)	AD	Clinical evidence (OMIM, Pubmed)
			Hypochondroplasia (146000)		
			LADD syndrome (149730)		
			Muenke syndrome (602849)		
			SADDAN (616482)		
			Thanatophoric dysplasia, type I (187600)		
			Thanatophoric dysplasia, type II (187601)		
*FIG4*	16873	NM_014845	Yunis-Varon syndrome (216340)	AR	Clinical evidence (OMIM, Pubmed)
*FLNB*	3755	NM_001457	Larsen syndrome (150250)	AD	Clinical evidence (OMIM, Pubmed)
*FREM1*	23399	NM_144966	Trigonocephaly 2 (614485)	AD	Clinical evidence (OMIM, Pubmed)
*GDF5*	4220	NM_000557	Multiple synostoses syndrome (610017)	AD	Clinical evidence (OMIM, Pubmed)
*GLI3*	4319	NM_000168	Greig cephalopolysyndactyly syndrome (175700)	AD	Clinical evidence (OMIM, Pubmed)
*IFT122*	13556	NM_052985	Cranioectodermal dysplasia 1 (218330)	AR	Clinical evidence (OMIM, Pubmed)
*IFT140*	29077	NM_014714	Short-rib thoracic dysplasia 9 with or without polydactyly (266920),	AR	Clinical evidence (OMIM, Pubmed)
*IFT43*	29669	NM_052873	Cranioectodermal dysplasia 3 (614099)	AR	Clinical evidence (OMIM, Pubmed)
*IFT52*	15901	NM_001303458	Short-rib thoracic dysplasia 16 with or without polydactyly (61702)	AR	Clinical evidence (OMIM, Pubmed)
*IHH*	5956	NM_002181	Copy number variations cause craniosynostosis Philadelphia type (185900)	AD	Clinical evidence (OMIM, Pubmed)
*IL11RA*	5967	NM_001142784	Craniosynostosis and dental anomalies (614188)	AR	Clinical evidence (OMIM, Pubmed)
*MASP1*	6901	NM_139125	3MC syndrome 1 (257920)	AR	Clinical evidence (OMIM, Pubmed)
*MEGF8*	3233	NM_001410	Carpenter syndrome 2 (614976)	AR	Clinical evidence (OMIM, Pubmed)
*MITF*	7105	NM_000248	Coloboma, osteopetrosis, microphthalmia, macrocephaly, albinism, and deafness syndrome (617306)	AR	Clinical evidence (OMIM, Pubmed)
*MSX2*	7392	NM_002449	Craniosynostosis, Boston type (604757)	AD	Clinical evidence (OMIM, Pubmed)
			Parietal foramina 1 (168500)		
*NOG*	7866	NM_005450	Multiple synostoses syndrome (186500)	AD	Clinical evidence (OMIM, Pubmed)
*P4HB*	8548	NM_000918	Cole-Carpenter syndrome (112240)	AD	Clinical evidence (OMIM, Pubmed)
*PAX3*	8617	NM_181457	Craniofacial-deafness-hand syndrome (122880)	AD	Clinical evidence (OMIM, Pubmed)
*POLR1C*	20194	NM_203290	Treacher-Collins syndrome 3 (248390)	AR	Clinical evidence (OMIM, Pubmed)
*POLR1D*	20422	NM_015972	Treacher-Collins syndrome 2 (613717)	AR/AD	Clinical evidence (OMIM, Pubmed)
*POR*	9208	NM_000941	Antley-Bixler syndrome (201750)	AR	Clinical evidence (OMIM, Pubmed)
*RAB23*	14263	NM_183227	Carpenter syndrome 1 (201000)	AR	Clinical evidence (OMIM, Pubmed)
*RECQL4*	9948	NM_004260	Baller-Gerold syndrome (218600),	AR	Clinical evidence (OMIM, Pubmed)
			Rothmund-Thomson syndrome (268400),		
			RAPADILINO syndrome (266280)		
*RSPRY1*	29420	NM_133368	Spondyloepimetaphyseal dysplasia, Faden-Alkuraya type (616723)	AR	Clinical evidence (OMIM, Pubmed)
*RUNX2*	10472	NM_001024630	Cleidocranial dysplasia (119600)	AD	Clinical evidence (OMIM, Pubmed)
*SF3B4*	10771	NM_005850	Acrofacial dysostosis, Nager type (154400)	AD	Clinical evidence (OMIM, Pubmed)
*SIX2*	10888	NM_016932	Frontonasal dysplasia, sagittal synostosis (n/a)	AD	Literature review (Pubmed)
*SKI*	10896	NM_003036	Shprintzen-Goldberg syndrome (182212)	AD	Clinical evidence (OMIM, Pubmed)
*SMAD6*	6772	NM_005585	{Craniosynostosis 7, susceptibility to}(617439)	AD	Literature review (OMIM, Pubmed)
*SMURF1*	16807	NM_001199847	Sporadic metopic craniosynostosis, craniofacial development		Literature review (Pubmed, MGI)
*SOX10*	11190	NM_006941	Craniofacial development		Literature review (Pubmed, MGI)
*SPRY1*	11269	NM_001258038	Craniofacial development		Literature review (Pubmed, MGI)
*SPRY4*	15533	NM_030964	Craniofacial development		Literature review (Pubmed, MGI)
*TCF12*	11623	NM_207036	Craniosynostosis 3 (615314)	AD	Clinical evidence (OMIM, Pubmed)
*TCOF1*	11654	NM_001135243	Treacher-Collins syndrome 1 (154500)	AD	Clinical evidence (OMIM, Pubmed)
*TGFBR1*	11772	NM_004612	Loeys-Dietz syndrome 1 (609192)	AD	Clinical evidence (OMIM, Pubmed)
*TGFBR2*	11773	NM_003242	Loeys-Dietz syndrome 2 (610168)	AD	Clinical evidence (OMIM, Pubmed)
*TTR*	12405	NM_000371	Maxillonasal dysplasia, Binder type	?	Clinical evidence (OMIM, Pubmed)
*TWIST1*	12428	NM_000474	Craniosynostosis 1(123100)	AD	Clinical evidence (OMIM, Pubmed)
			Robinow-Sorauf syndrome (180750)		
			Saethre-Chotzen syndrome (181400)		
*WDR19*	18340	NM_025132	Cranioectodermal dysplasia 4 (614378)	AR	Clinical evidence (OMIM, Pubmed)
*WDR35*	29250	NM_001006657	Cranioectodermal dysplasia 2 (613610)	AR	Clinical evidence (OMIM, Pubmed)
			Short-rib thoracic dysplasia 7 with or without polydactyly (614091)		
*ZIC1*	12872	NM_003412	Craniosynostosis 6 (616602)	AD	Clinical evidence (OMIM, Pubmed)

AD – autosomal dominant, AR – autosomal recessive, XD – X-linked disorder.

**Table 2:** Common SNVs associated with non-syndromic sagittal craniosynostosis included in craniosynostosis-associated genes panel (based on Justice *et al*.^46^).

SNV	Gene	Genomic region	Description
rs1009355	BBS9	Chr7:33218763	common intron variant; NM_198428.2:c.442 + 1560T > A
rs10254116	BBS9	Chr7:33237489	common intron variant, NM_198428.2:c.442 + 20286T > C
rs10262453	BBS9	Chr7:33256039	common intron variant, NM_198428.2:c.442 + 38836A > C
rs1420154	BBS9	Chr7:33290931	common intron variant, NM_198428.2:c.443-5917G > A
rs142092	n/a	Chr20:7093432	common genomic variant, NC_000020.10:g.7093432T > C
rs179753	LINC01428	Chr20:7151968	common intron variant, NR_110609.1:n.298 + 12022C > T
rs1884302	n/a	Chr20: 7106289	common genomic variant, NC_000020.10:g.7106289T > C
rs4140470	LINC01428	Chr20:14371737	common intron variant, NR_110609.1:n.164 + 14997T > C
rs6054814	LINC01428	Chr20:7198501	common intron variant, NR_110609.1:n.164 + 23975C > A
rs6107929	n/a	Chr20:7121672	common intron variant, NC_000020.10:g.7121672A > G
rs6140226	LINC01428	Chr20: 7226483	common intron variant, NR_110609.1:n.117-3960G > A

### Quality control and coverage estimation

**Figure 1:**
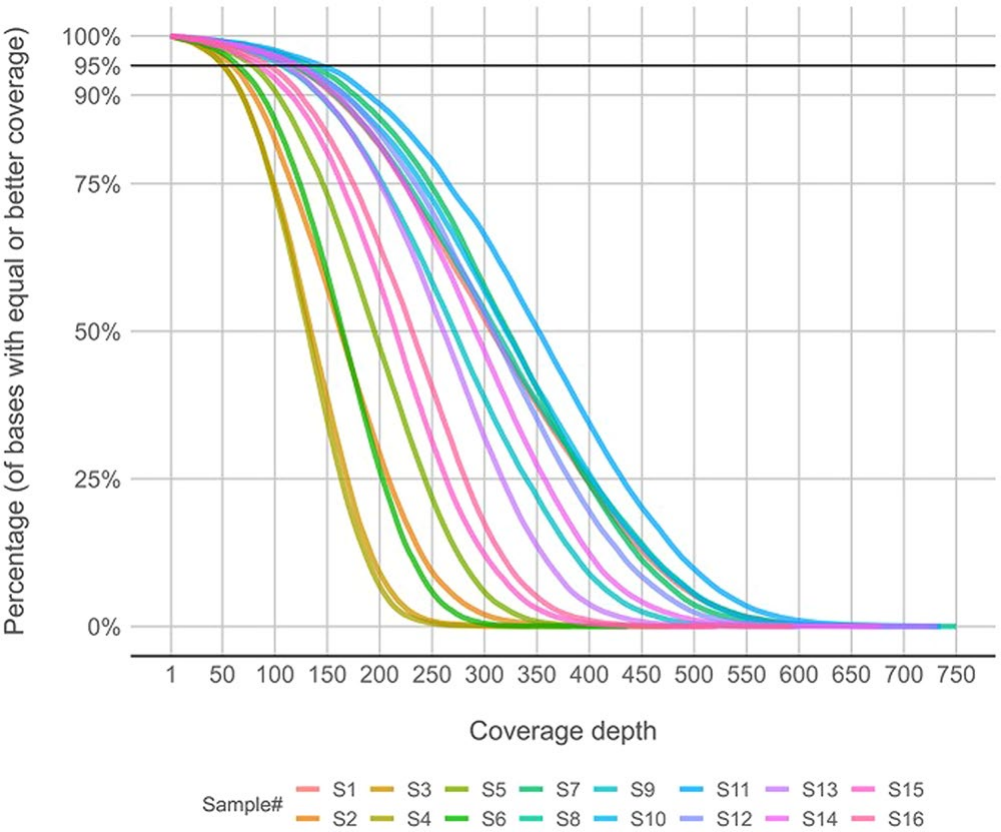
Comparison of per-base coverage depth for all samples. Additional horizontal line indicates 95% of total bases in panel target regions.

In each sample the estimated coverage exceeded 50 reads for over 95% of the target gene sequence (see Fig. 1). Mean coverages for the analysed genes and single nucleotide variants (SNVs) are summarised in Supplementary Materials (see Supplementary 1). There were marked discrepancies among the mean coverages of different samples, ranging from 129 in sample 3 to 337 in sample 11, with an average coverage of 240 reads calculated per gene. Across individual genes, *SMAD6* had the lowest average coverage of 133, while *POLR1D* was relatively best covered (321 on average).

### Identification and evaluation of candidate variants

**Figure 2:**
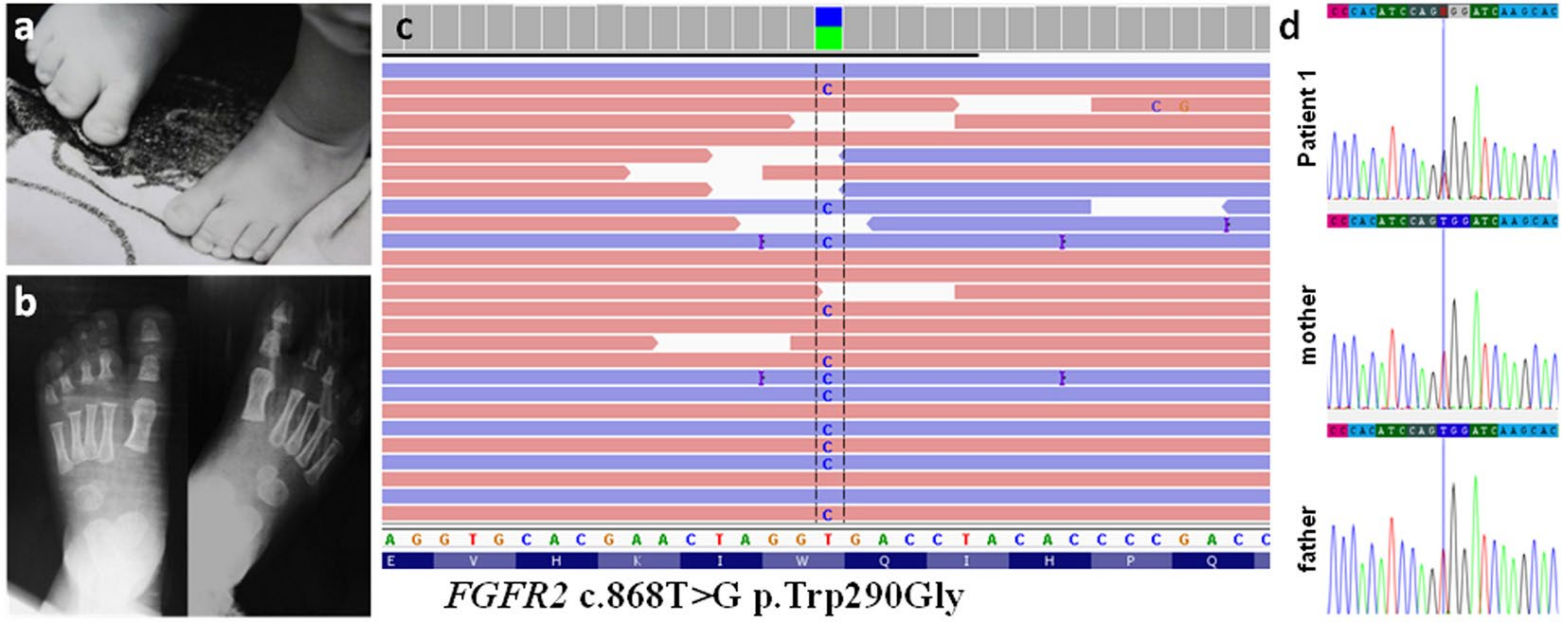
Clinical characteristics at the age of 12 months (**a,b**) as well as molecular results of Patient 1 (**c,d**). **Patient 1**, in addition to sagittal craniosynostosis, maxillary hypoplasia, high palate and proptosis, presented with broad halluces and skin syndactyly of 2^nd^ and 3^rd^ toes **(a)**. X-ray of the feet showed small hypoplastic middle phalanges of all toes, relative widening of 1^st^ metatarsals and broadening of phalangeal bones forming halluces, and no bone syndactyly of 2^nd^ and 3^rd^ toes (**b**) Representation of the heterozygous *FGFR2* deleterious variant c.868T > G p.Trp290Gly detected in Patient 1 by means of targeted next-generation sequencing **(c)** and validation studies of the proband and parental testing of the *FGFR2* gene with the use of Sanger sequencing **(d)**. Pathogenic variant c.868T > G p.Trp290Gly was confirmed in the index case and excluded in his unaffected parents, clearly indicating a de novo occurrence.

After sequencing of all 16 DNA samples on the Ion Torrent S5 system and completing the alignments, we assessed variant quality using multiple criteria (see Methods) and predicted the significance of individual variants. During the quality control, out of 2565 called variants, 87 (3.4%) were dropped as artefacts. In three cases, we detected the variants definitely causative for the patients’ phenotypes. Patient 1 was suspected of Pfeiffer syndrome, based on the clinical assessment. His phenotype involved sagittal CS, maxillary hypoplasia, high palate, proptosis, broad halluces, and skin syndactyly of 2^nd^ and 3^rd^ toes. X-ray examination of the feet showed hypoplastic middle phalanges of all toes and the relative widening of 1^st^ metatarsals as well as broadening of all bones forming the halluces (see Fig. 2a,b). Upon NGS analysis we found a pathogenic heterozygous variant in *FGFR2* gene NM_000141.4:c.868T > G, NP_000132.3:p.Trp290Gly (HGMD: CM950464, ClinVar: 13284) (see Fig. 2c,d). Pathogenic variant was confirmed by means of Sanger sequencing in the index case and excluded in his unaffected parents, clearly indicating a de novo occurrence.

**Figure 3:**
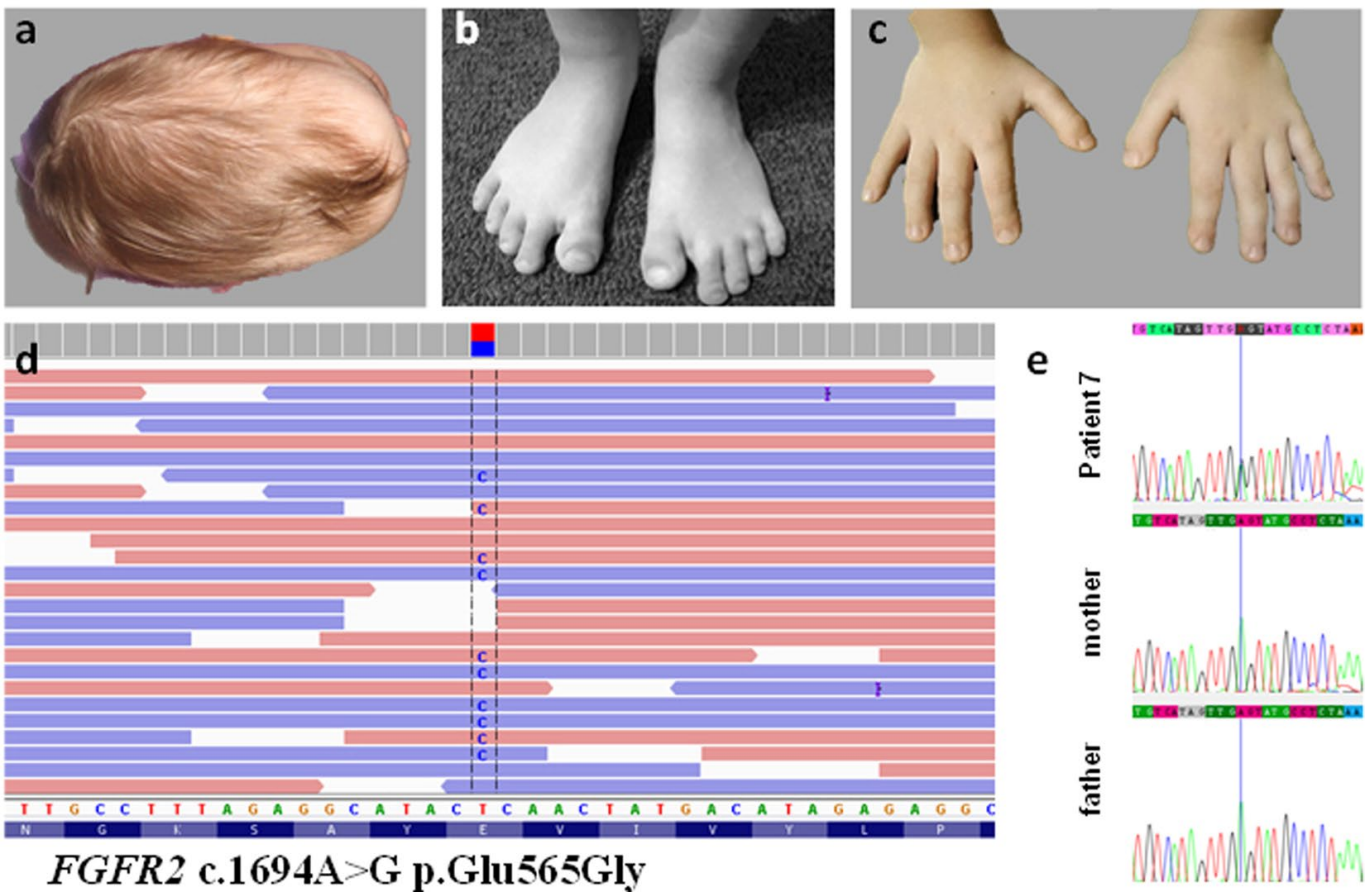
Clinical characteristics at the age of 2 years (**a**) and 6 years (**b,c**) as well as molecular results of Patient 7 (**d,e**). **Patient 7** presented with complex craniosynostosis involving sagittal and bilateral coronal synostosis, dolichocephaly, macrocephaly, prominent forehead **(a)**, flat face, proptosis (full facial picture not shown),brachydactyly and broad halluces **(b,c)**. Representation of the heterozygous *FGFR2* deleterious variant c.1694A > G p.Glu565Gly unraveled in Patient 7 by means of targeted next-generation sequencing **(d)** and validation studies of the proband and parental testing of the *FGFR2* gene with the use of Sanger sequencing **(e)**. Pathogenic variant c.1694A > G p.Glu565Gly was confirmed in the index case and excluded in his unaffected parents, clearly indicating a de novo occurrence.

As female Patient 7 presented with complex CS involving sagittal and bilateral coronal synostosis, dolichocephaly, macrocephaly, prominent forehead, flat facial profile, proptosis, brachydactyly and broad halluces, clinical diagnosis also matched Pfeiffer syndrome (see Fig. 3a–c). At a molecular level, we identified a pathogenic heterozygous variant in *FGFR2* NM_000141.4: c.1694A > G, NP_000132.3:p.Glu565Gly (HGMD: CM043278, ClinVar: 374823) (see Fig. 3d,e). Pathogenic variant was confirmed by means of Sanger sequencing in the index case and excluded in his unaffected parents, clearly indicating a de novo occurrence.

**Figure 4:**
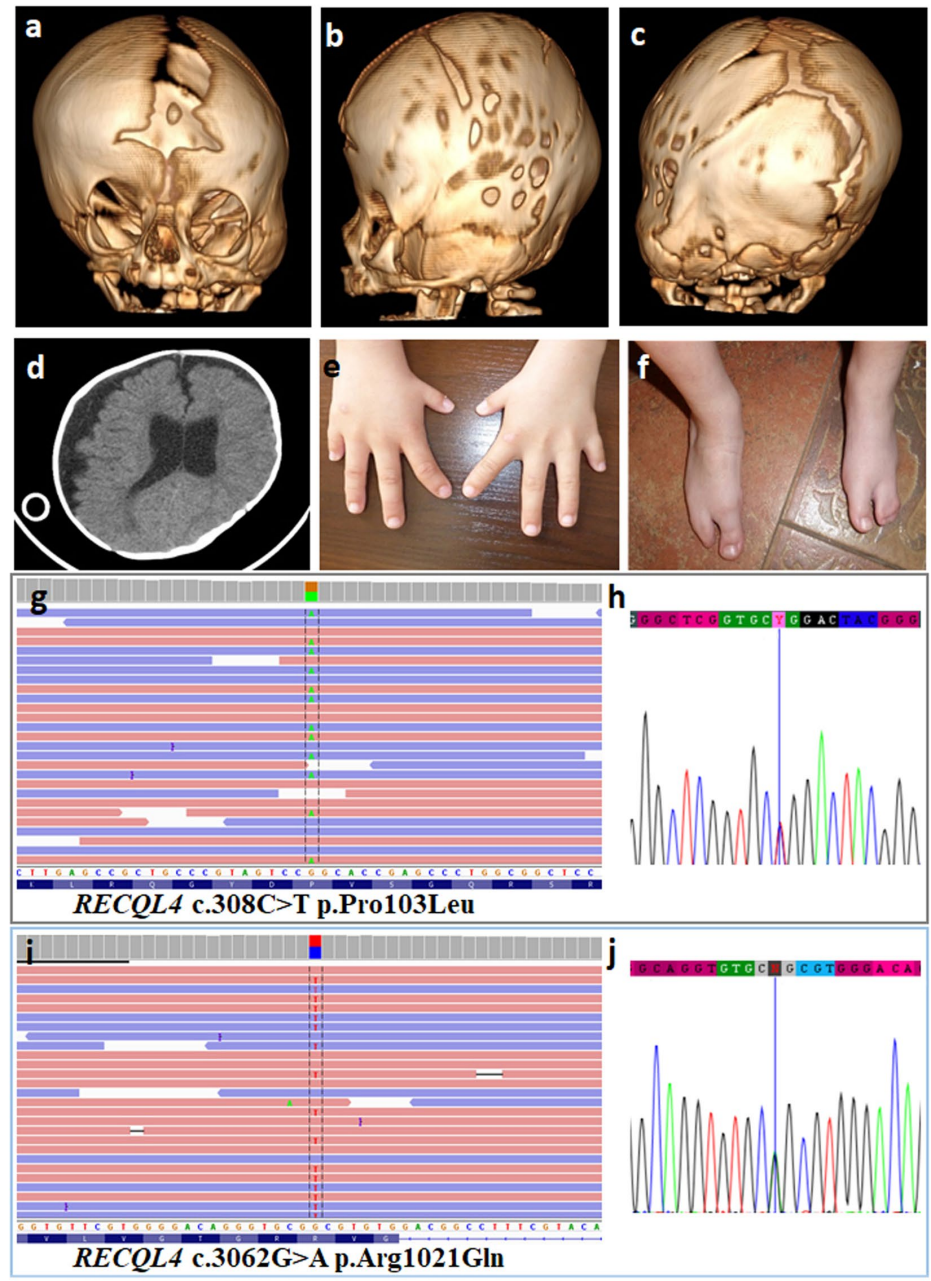
Clinical characteristics of Patient 15 at the age of 11 **(a–d)** and 9.5 years **(e,f)** as well as molecular results of the patient (g-j). **Patient 15** presented with complex craniosynostosis composed of bilateral coronal synostosis (complete right-sided and partial left-sided) as well as partial left-sided lambdoid synostosis shown in 3D modelling of the skull **(a–c)**. CT scan of the head **(d)**. Coronal sutures are prematurely fused. The right coronal suture is completely fused **(a)**, while the left one is only partially fused **(b)**; consequently, there is marked enlargement of the anterior fontanelle and widening of the sagittal suture, **(a,c)**. Asymmetry of the skull and brain, including lateral ventricles, and enlargement of left subarachnoid space seen on horizontal section **(d)**. Limb defect clinically recognized as bilateral split foot malformation with syndactyly of the remaining toes, extremely short and hypoplastic thumbs and 5^th^ fingers, short 5^th^ metacarpals and valgus deformity of the right 2^nd^ finger **(e,f)**. Representation of the compound heterozygous *RECQL4* deleterious variants c.308C > T p.Pro103Leu and 3062G > A p.Arg1021Gln detected in Patient 15 by means of targeted next-generation sequencing **(g,i)**. Both pathogenic variants were confirmed with the use of Sanger sequencing **(h,j)**.

In Patient 15, affected by complex CS the defect was composed of bilateral coronal synostosis (complete right-sided and partial left-sided) as well as partial left-sided lambdoid synostosis, marked craniofacial asymmetry, hearing impairment, scoliosis, bilateral split foot malformation with syndactyly of the remaining postaxial toes, extremely short and hypoplastic thumbs and 5^th^ fingers, short 5^th^ metacarpals and valgus deformity of the right 2^nd^ finger we detected two pathogenic variants in *RECQL4* gene NM_004260.3:c.308C > T NP_004251.3, p.Pro103Leu (HGMD: CM033805, Clinvar: 239755) and c.3062G > A, p.Arg1021Gln (HGMD: CM033810, ClinVar: 135147) (see Fig. 4a–j). The two variants were confirmed by Sanger sequencing.

Intellectual development was normal in all three presented patients.

## Discussion

Craniosynostoses encompass a group of distinct, clinically variable phenotypes. Since the first dysmorphological description of the disease by Wheaton in late 19^th^ century, researchers have been extensively investigating the molecular background of CS^25^. The first causative gene for this condition was identified by Jabs *et al*. in 1993, who described a pathogenic variant within *MSX2* in a family affected by autosomal dominant CS^26^. In the next four years, a few novel genes – *FGFR1*, *FGFR2*, *FGFR3*, *TWIST1* – have been linked to premature fusion of the cranial sutures^27–31^. Recently, the development of high-throughput NGS-based strategies allowed for unravelling of the molecular basis of the condition at an unprecedented scale, as it happened for newly described variants, e.g. within *ERF, SMAD6*, and *TCF12*^32–34^. However, in a significant percentage of cases, pathogenesis of the craniosynostosis is still unknown or only partially understood^21,35,36^. Thus, there is an unquestionable need of further research by means of WES or WGS to find novel genes or non-coding variants responsible for the development of CS in humans. In the diagnostic setting, however NGS-based panel approach appears to be a sufficient solution for mutational screening of all known causative genes or variants.

Here, we proved that a custom NGS panel designed by us represents a useful and effective tool in the molecular diagnostics of patients with CS. We investigated 16 unrelated patients and provided a diagnosis at a molecular level for 3 (19%) of them, demonstrating the high coverage and high quality of the sequencing data at the same time. In Patient 1 and 7, the pathogenic variants were previously described in individuals affected either by Crouzon or Pfeiffer syndromes. Clinical evaluation of our patients was consistent with the diagnosis of Pfeiffer syndrome^37–39^. Interestingly, the variant detected in Patient 7, who presented with complex CS, macrocephaly, prominent forehead, flat face, proptosis, and broad halluces may not only give rise to Pfeiffer or Crouzon syndromes with normal intellectual development, but also to a more severe cloverleaf skull phenotype with an early demise^40^. A broad phenotypic spectrum resulting from the same pathogenic variant suggests a possibility of other yet unidentified genetic or environmental modifiers, as indicated by Oldridge and colleagues^41^.

Patient 15 carried two pathogenic *RECQL4* variants, both described as causative for Rothmund-Thomson syndrome (RTS) in osteosarcoma association study^42^. Both variants are very likely to occur in patient 15 in trans orientation, as they were identified in heterozygous state in two different probands^42^. Additionally, both p.Arg1021Pro and p.Pro103Leu *RECQL4* alterations represent rare or extremely rare founder mutations already described in GnomAD/ExAC database. The variant p.Arg1021Pro was annotated in heterozygosity in 2 out of almost 280 thousand control alleles, while the variant p.Pro103Leu in 168 out of about 280 thousand alleles, clearly suggesting that those two SNVs do not represent a common haplotype. Importantly, none of the variants was found in homozygosity in a control healthy population, additionally indicating high likelihood of their pathogenicity. Unfortunately, we were unable to check the parental status for the RECQL4 mutations, as the parents disagree to undergo genetic testing. Interestingly, mutations within *RECQL4* gene are linked to three distinct autosomal recessive conditions with overlapping phenotypes, i.e. Rothmund-Thomson, RAPADILINO, and Baller-Gerold syndrome (BGS), but only the last disorder comprises hearing loss in its clinical spectrum^43–45^. Considering lack of poikiloderma and the presence of hypoacusis in Patient 15, our final diagnosis was BGS. With clinical and molecular data presented here, we have broadened the phenotypic spectrum of previously reported *RECQL4* alterations that may give rise to either RTS or BGS phenotype.

Although we confirmed the molecular diagnosis only in 3 out of 16 probands, the diagnostic yield of 19% is equal to some of the published research reports (21%)^21^. Importantly, we analysed only the patients with negative results of Sanger sequencing for *TWIST1* alterations and the most common pathogenic hot-spot variants of *FGFR1, FGFR2, FGFR3*. Consequently, our diagnostic score was significantly lower than e.g. 62% reported by Paumard-Hernández *et al*., who did not perform any molecular prescreening in the analysed individuals^22^.

Our study, in which a cohort of CS patients was utilised as an example, demonstrated the usefulness of targeted gene panel sequencing in the diagnostics of complex, genetically heterogeneous conditions. To our knowledge, we were the first to adapt SureSelect hybridisation-based enrichment protocol for the sequencing on the Ion Torrent S5 platform, which is intended to work preferably with amplicon-based panels (f.e. AmpliSeq® Thermo Fisher Scientific). Agilent hybridisation technology has not been previously used on Ion Torrent S5 equipment, hence the total cost of the analysis is higher than the standard ThermoFisher Scientific procedure. This is due to the fact that our experiment was focused on obtaining the most optimal quality of sequencing data and not on the cost reduction. The estimated price for the analysis was about 1.7 times higher per sample compared to the standard procedure, but optimisation of cost is certainly achievable. Although SureSelect hybridisation-based protocol is about 1.5 times more time consuming and represents a costlier alternative in comparison to amplicon-based approach, it provides several advantages, especially in the diagnostic setting, such as reduction of PCR-related edge artefacts, better and more exact matching of hybridisation probes. Consequently, it allows for obtaining higher specificity of the amplified region.

With the approach presented here, we achieved high molarity of both pooled libraries (756 and 525 pmol/l) and exceeded coverage of 50 reads in each sample for over 95% of the target gene sequences (173.794 kb), as well as an average coverage of 240 reads per gene across all samples. Importantly, we obtained full coverage for all of the exons within *FGFR2*, which was not possible in amplicon-based protocols (e.g. Ampliseq®, Thermo Fisher Scientific). Moreover, we were able to include all of the listed genes, which was impossible using even an advanced made-to order option in Ion AmpliSeq Designer tool (Thermo Fisher Scientific).

In conclusion, we successfully adapted hybridisation-based SureSelect enrichment protocol for the Ion Torrent S5 platform, demonstrating that an alternative enrichment strategy for library preparations can be applied prior to sequencing on the Ion Torrent S5. Additionally, we proved the efficiency and clinical utility of the designed gene panel in the genetic testing of patients affected by variable CS.

## Methods

All procedures involving human participants were performed in accordance with the ethical standards of the institutional and/or national research committee and with the 1964 Helsinki declaration and its later amendments or comparable ethical standards. Ethics approval was granted by the Institutional Review Board of Poznan University of Medical Sciences (no 742/17 obtained on 22^th^ June 2017). All patients and their parents agreed to participate in this study. This research involved human participants under the age of 18 years. Hence we obtained informed consents from parents and/or legal guardians. We present information or images that could lead to the identification of study participants. Accordingly, a specific consent has also been obtained from all parents and/or legal guardians for publication of identifying information/images in an online open-access publication.

### Sample preparation

We extracted genomic DNA from the peripheral blood lymphocytes using the MagCore® HF16 Automated Nucleic Acid Extractor and quantified each gDNA using the Agilent Technologies TapeStation 4200 and Genomic DNA ScreenTape systems. The custom panel designed by us comprised 61 genes and 11 SNPs (see Tables 1 and 2) known to be involved in the development of craniofacial malformations, including craniosynostosis, in human and mouse. Prior to NGS, we performed targeted molecular screening of all patients for the common hot-spot mutations located in exon 7 of *FGFR1* (c.755C > G p.Pro252Arg), exons 7 and 8 of *FGFR2* (c.755C < G p.Ser252Trp and c.758C > G p.Pro253Arg), and exon 7 of *FGFR3* (c.749C > G p.Pro250Arg) as well as we analysed the entire coding sequence of *TWIST1* by means of Sanger sequencing. For NGS, we used high-molecular DNA with a range of DNA Integrity Number (DIN) 6.8 to 9.6. In the next step, Ion Shear Plus reagent (Thermo Fisher Scientific) cut each genomic DNA sample (1 μg) into fragments of 50–250 bp. To obtain approximately 130 bp peaks, we adjusted the time of incubation at 37 °C to 50 minutes (step 1). Afterwards, we ligated each sample with Ion P1 Adapter and Ion Express barcode (Thermo Fisher Scientific). The ligation was as follows: 15 minutes at 25 °C, 5 minutes at 72 °C. We used a thermal cycler without a heated lid (40 °C) (step 2). Next, we proceeded to amplification of the adapter-ligated libraries through PCR reaction. To obtain an adequate yield for subsequent capture without introducing bias or non-specific products, we performed pre-capture PCR with Herculase II Fusion DNA Polymerase (Agilent Technologies) consisted of 8 cycles (step 3). After steps 1–3 we purified each sample with the use of Agencourt AMPure XP beads (Beckman Coulter Genomics), whereas after steps 1 and 3 we assessed the quality and quantity of samples on 4200 TapeStation using D1000 ScreenTape system (Agilent Technologies). In the first measurement of the samples (step 1) the obtained concentrations were between 0.229 and 6.39 ng/µl, while in the final phase (step 3) concentrations ranged from 32.2 to 69.4 ng/µl.

### Hybridisation and capture

We prepared the NGS libraries for Ion Torrent S5 platform using hybridised capture-based target enrichment approach (SureSelect) developed by Agilent Technologies. We performed hybridisation of 750 ng in 3.4 μl of each genomic DNA library using SureSelect Target Enrichment Reagent Kit according to manufacturer’s protocol for <3 Mb capture libraries. After 17 hours of hybridisation at 65 °C, we captured the targeted molecules on streptavidin-coated magnetic beads (Dynabeads MyOne Streptavidin T1).

### Post-hybridisation amplification and sample processing for multiplexed sequencing

We amplified purified SureSelect-enriched DNA libraries and non-template control through PCR (11 cycles) with the use of Herculase II Fusion Polymerase. Before assessing DNA quality and quantity with High Sensitivity DNA Assay on TapeStation System, we purified each sample using AMPure XP beads. Based on the evaluated concentration of SureSelect-enriched DNA libraries, we calculated the amount of each sample to be included in the pool using the following formula: volume of barcoded sample: V(f)xC(f)/nxC(i). V(f) is the final required/needed volume of the pool (20 µl), C(f) is the initial concentration of all SureSelect-enriched DNA libraries in the pool, n is the number of samples to be combined, and C(i) is the initial concentration of each barcoded sample. To avoid the presence of additional fragments in each library, we size-selected our pools by agarose gel electrophoresis using the integrated E-Gel system (Thermo Fisher Scientific), purified them using Agencourt AMPure XP beads (Beckman Coulter Genomics) and finally validated using High sensitivity DNA assay on 4200 TapeStation. The molarity of pooled libraries was 756 and 525 pmol/l, respectively (see Supplementary Fig. 1). Since the Ion Chef requires concentration of a loaded pooled library to be 50 pM, we diluted our samples using low TE buffer.

### Emulsion PCR and sequencing

We subjected 25 µl pooled libraries to emulsion PCR on the Ion Chef Instrument with the use of the Ion 520™&530™ Kit, according to the manufacturer’s protocol. Finally, we sequenced each loaded Ion 520™ chip on the Ion Torrent S5 System with the use of recommended reagents.

### Sanger sequencing

We confirmed pathogenic variants using a conventional Sanger sequencing. We designed specific primers for the amplification using Primer3 tool (see Supplementary Table 1) and carried out the PCR reactions in a mixture containing the following substrates: DNA, 10 × PCR Premix J buffer, primers, H_2_O and DNA polymerase. The PCR products were purified with Exonuclease I and shrimp alkaline phosphatase. Sequencing of the PCR product was carried out using dye-terminator chemistry (kit v.3, ABI 3130XL) and run on automated sequencer Applied Biosystems Prism 3700 DNA Analyser.

### Bioinformatic analysis

Reads were initially demultiplexed and aligned to GrCh37 human reference sequence using the TorrentBrowser 5.0.4 software (Thermo Fisher Scientific) running as embedded instance within Ion Torrent S5 sequencer. The resulting alignment BAMs were further processed using IonReporter 5.2 pipeline (Thermo Fisher Scientific), which incorporated variant calling. Estimation of coverage for individual genes/positions was conducted via bedtools 2.27.1 (*coverage* subcommand) against a BED file defining coding parts of canonical transcripts (RefSeq mapped on UCSC hg19 reference; 5 bp padding around each exon included; see Supplementary 2). Variant quality control was assessed based on a fourfold metric (read depth - greater than or equal to 20, strandedness - no more than 4:1 difference in reporting of the variant on opposite strands, PHRED quality of over 30, and variant proportion of not less than 15% of total reads). The existence of potentially significant variants was further reassessed through manual inspection of aligned reads in IGV 2.4 software.

Available clinical significance annotation was assessed in real-time from Human Gene Mutation Database Professional (https://portal.biobase-international.com/hgmd/pro/), ClinVar (https://www.ncbi.nlm.nih.gov/clinvar/) and dbSNP (https://www.ncbi.nlm.nih.gov/snp) on 21/03/2018. The predictions for SIFT, PolyPhen and PhyloP (46-way) tools were retrieved from the IonReporter result files (tab-separated files). Frequency data was provided by Ensembl/VEP (software version 91, database version 91); additionally GNOMAD database was queried for homozygosity/heterozygosity of individual variants (http://gnomad.broadinstitute.org; version 2.0.2 of both exome and genome subsets; accessed on 21/03/2018 using tabix 1.5 software). The outcomes for Combined Annotation Dependent Depletion were obtained from CADD webserver (version 1.3, https://cadd.gs.washington.edu; accessed on 21/03/2018). MutationTaster results were obtained using ‘query chromosomal position’ options of the public webserver (http://www.mutationtaster.org; accessed on 21/03/2018). SnpEff prediction of variant consequences was obtained using local installation of SnpEff 4.3t with default databases for hg19 reference. The effect of substitutions on splicing was assessed using ADA + RF predictors available through dbNSFP v.3.5a (dbscSNV 1.1 dataset).

Visualisation of variants within gene/protein sequence context was done using R/Bioconductor package trackViewer (1.16.1, ran in R 3.4.1).

## Supplementary information

Supplementary information.

Figure 1 Table 1.

Supplementary 1.

Supplementary 2.

## Data Availability

BAMs files were submitted on Sequence Read Archive (SRA) NCBI (SRA accession: PRJNA597426 https://www.ncbi.nlm.nih.gov/sra/PRJNA597426; release date: 2020-07-01).
