# Supplementary material for: Adapting SureSelect enrichment protocol to the Ion Torrent S5 platform in molecular diagnostics of craniosynostosis
Source: Sci Rep. 2020 Mar 5;10:4159. doi: 10.1038/s41598-020-61048-5 (PMC7058001; doi:10.1038/s41598-020-61048-5)
Supplement: Supplementary file 1 — Supplementary information. [file 41598_2020_61048_MOESM1_ESM.docx]

**Adapting SureSelect enrichment protocol on the Ion Torrent S5 platform in molecular diagnostics of craniosynostosis**

Ewelina Bukowska-Olech^1^, Delfina Popiel^2^, Grzegorz Koczyk^2,3^ Anna Sowińska-Seidler^1^, Magdalena Socha^1^, Bartosz Wojciechowicz^4^, Adam Dawidziuk^2^, Dawid Larysz^5^, Aleksander Jamsheer^1*^

**Figure 1** The electropherograms presenting size peaks of pooled libraries.

The molarity of each pool was evaluated based on presented electropherograms – 756 pmol/l **(a)** and 525 pmol/l **(b)**

**Supplementary 1** Mean coverages for the analysed genes and single nucleotide variants (SNVs)

**Supplementary 2** Coding parts definition

**Table 1** Primers sequences used for Sanger sequencing
