# Supplementary material for: Adapting SureSelect enrichment protocol to the Ion Torrent S5 platform in molecular diagnostics of craniosynostosis
Source: Sci Rep. 2020 Mar 5;10:4159. doi: 10.1038/s41598-020-61048-5 (PMC7058001; doi:10.1038/s41598-020-61048-5)
Supplement: Supplementary file 2 — Figure 1 Table 1. [file 41598_2020_61048_MOESM2_ESM.docx]

**Adapting SureSelect enrichment protocol on the Ion Torrent S5 platform in molecular diagnostics of craniosynostosis**

Ewelina Bukowska-Olech^1^, Delfina Popiel^2^, Grzegorz Koczyk^2,3^ Anna Sowińska-Seidler^1^, Magdalena Socha^1^, Bartosz Wojciechowicz^4^, Adam Dawidziuk^2^, Dawid Larysz^5^, Aleksander Jamsheer^1*^

**Figure 1**

^
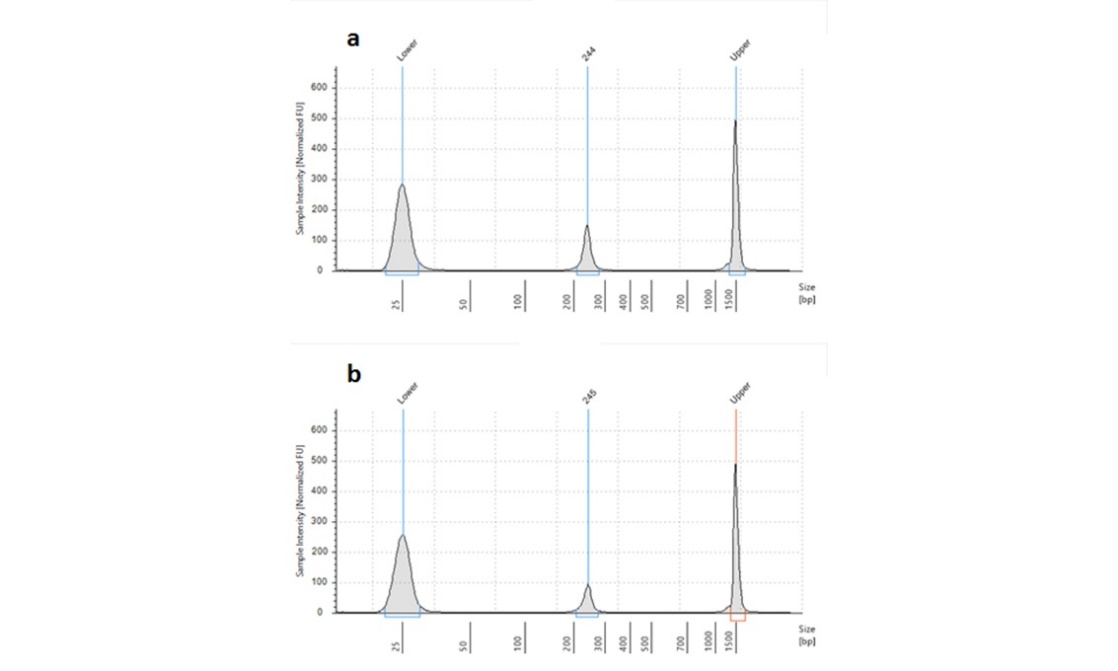
^

| **Gene** | **Exon** | **Forward primer sequence 5’-3’** | **Reverse primer sequence 5’-3’** |
| --- | --- | --- | --- |
| *FGFR2* | 7 | AGGTCACTGACAGCCCTCTG | CATGAAGGAGACCCCAGTTG |
| *FGFR2* | 13 | GTTTTGCTGAATTGCCCAAG | CCACCCAGCCAAGTAGAATG |
| *RECQL4* | 4 | CTCCTCCCACTTCCCTGTTT | AGTCCCCACGCTCAATTGTA |
| *RECQL4* | 20 | cctgcctgcatctgacatg | ggaggagcctgtcagagc |

**Table 1** Primers sequences used for Sanger sequencing
